# Models attempting to quantify the relationship between drug development and financial return are missing a key element: the effect on post-approval research

**DOI:** 10.1093/haschl/qxaf188

**Published:** 2025-10-21

**Authors:** Dan Crippen, Kirsten Axelsen

**Affiliations:** Board Advisor and Former Congressional Budget Office Director, Senior Policy Advisor DLA Piper, New York, NY 10029, United States; Board Advisor and Former Congressional Budget Office Director, Senior Policy Advisor DLA Piper, New York, NY 10029, United States

**Keywords:** biopharmaceutical, Inflation Reduction Act, Congressional Budget Office, clinical development

## Abstract

Changes in biopharmaceutical policy, specifically the Inflation Reduction Act (IRA), introduced administrative drug price setting in the U.S., prompting questions about the impact on drug development of this and future policies under consideration. Existing models used to inform policymakers, such as those from the Congressional Budget Office (CBO), attempted to quantify the relationship between investment and financial return but overlooked the effect on post-approval research. This research, essential for expanding drug indications and demonstrating efficacy in new populations, is often pursued years after initial approval, at the time when IRA price controls take effect. As a result, the expected financial return from secondary indications is diminished, potentially discouraging investment in post-market studies. This commentary emphasizes the importance of models that incorporate the impact of policy on both new and post-approval drug development. Without such analysis, policymakers risk underestimating the broader consequences. Given the significant role post-approval research plays in improving health outcomes, particularly for chronic disease, its exclusion from policy impact models is a notable gap. We urge the research community to generate evidence that informs more comprehensive modeling, ensuring that future policy decisions support investment in the entire lifecycle of drug development.

Administrative price setting for drugs is being implemented in the U.S., with other policies to reduce prices under consideration. However, the implications on drug development are poorly described by existing policy impact models. It is possible, even plausible, that no model can be developed to reliably describe the inputs that affect investment into drug development and its relationship to the financial return from that activity. Here, we consider one notable, and fixable, limitation in the forecast most widely used by policymakers. It lacks an estimate of the impact of biopharmaceutical policy change on post-approval research for existing drugs.

Post-approval research is used to develop new indications or to demonstrate safety and efficacy in new populations. Without this evidence, health insurance plans may deny coverage for expanded uses. Moreover, the health system loses out when this information is not systematically collected. Models developed by the Congressional Budget Office (CBO) to inform policymakers should include an assessment of the impact of policy on both new and post-market drug development. Policymakers should use caution when interpreting models, recognize this significant limitation, and appreciate that no model is an accurate forecast of a complex policy change.

## Background

The Inflation Reduction Act (IRA) was passed in 2022, granting the federal government the authority to directly regulate drug prices in Medicare while making other changes in cost sharing and subsidies. The implementation of the IRA has progressed rapidly without a thorough understanding of its impact on drug development. Other drug-focused policies, including tariffs and international price referencing, are under consideration, while understanding their impact on drug development, and, on health is limited.

This commentary considers the CBO model used to estimate the impact of biopharmaceutical policy on drug development. The CBO built, iterated, and improved its model and called for input on evidence and methods.^1^ We note a limitation to the CBO approach still to be addressed: it lacks assessment of the impact of biopharmaceutical policy change on post-approval drug development. Their model considers only new drug development. We note the importance of investment in post-market indication expansion, which should be considered in policymaking.

## Controlling the price of medicines vs investment in drug development

Two priorities–lower drug costs and investment in drug development—compete for consideration in policy debates. Currently, this debate includes (1) changes to the IRA; (2) alignment of U.S. prices to foreign countries; (3) changes in intellectual property protection; and (4) tariffs on imported drugs and drug precursors, among others. These all impact the financial reward from drug development.

Analysts have weighed in on the debate by providing estimates. These build on evidence developed over the past 20 years that describes the relationship between investment in clinical development and pharmaceutical market return and includes regression and simulation models evaluating the U.S. and foreign markets.^2^ Estimates by the CBO and others of the number of drugs foregone by the imposition of the price controls in the IRA range from 13 fewer new drugs being developed over 30 years to 139 drugs not developed over 10 years.^3,4^

In the IRA, medicines selected for drug price negotiation must have been on the market for 7 years for small molecules or 11 years for biologics, going into effect 2 years later. Due to the timing, selected drugs see less revenue when additional indications and post-market studies are often pursued, affecting the expectation of a return on investment.^5^ While the CBO does not consider post-market development, others have described how the IRA could affect this type of research into new uses for approved medicines.^6,7^

## The value of post-approval research in secondary drug development

Identifying a new mechanism that modifies a disease through clinical research is expensive and time-consuming; post-market indications are critical to expanding the value of approved medicines and identifying new ways a drug can be used. Between 2000 and 2023, 51% of newly approved drugs had one or more post-approval expansions, with the highest numbers of post-approval expansions occurring in oncology, neurology, and infectious disease.^8^

Recent examples of valuable post-market innovation include long-acting injectable medicines that can help improve adherence (eg, lenacapavir for HIV), drugs approved for earlier stages of cancer after previously being approved for more advanced diseases (eg, acalabrutinib for leukemia and lymphoma), and drugs demonstrated safe and effective for use in children that were previously developed for adults (eg, aflibercept for pre-term infants with eye disease, initially approved for macular degeneration).

## Why does it matter?

If a drug is selected for IRA price negotiation, the expectation of profitability in the latter years post-approval is reduced, particularly if that drug is expected to be used by Medicare beneficiaries. There are two implications: first, the expected financial return from developing a new drug is reduced, which reduces the likelihood that a drug will be developed, and second, the financial return from post-market investment is diminished, thus reducing the likelihood of investment in secondary indications.

Post-market research, including additional clinical studies, can broaden the use of existing drugs to improve human health by (1) identifying new indications; (2) establishing efficacy in a new population, such as children; (3) evaluating the drug for different disease states; and (4) developing new ways to administer the drug.

Approved drugs are often used for “off-label,” meaning a physician prescribes a medicine for a use other than the approved indication or population. However, health plans can and often do deny coverage for off-label use. Moreover, post-market studies provide information to others in the healthcare system about other uses of approved medicines. Policies that deter investment in post-market research limit access to information and treatment.

## Conclusion

Chronic disease treatment consumes most of the healthcare spending in the U.S. while producing worse health outcomes than other countries. This means that we must do much better at both prevention and treatment, including the use of medicine.^9^ Until alternative, effective treatments, including lifestyle changes, are developed, it is essential to understand how policies affect investment in clinical studies for both new and existing medicines.

Biopharmaceutical innovation has meaningfully contributed to longer lifespans and better health in the U.S. and around the world. Both new drugs and post-approval research to identify secondary indications of existing drugs will continue to play a significant role in maintaining health. The assessment that informs policymakers, the CBO models, does not consider the impact of policy change on investment in new uses of existing drugs. This can be addressed.

It remains uncertain whether any model, simulation, regression, or otherwise, can adequately capture all the complex effects of biopharmaceutical policy change, or whether that level of precision is even a reasonable expectation. At a minimum, analysis should account for the impact on post-approval clinical development.

## Supplementary Material

qxaf188_Supplementary_Data

## References

[1] Congressional Budget Office Blog . Phil Swagel “A Call for New Research in the Area of New Drug Development,” December 2023. Accessed September 30, 2025. https://www.cbo.gov/publication/59818.

[2] Innovation Technology and Innovation Foundation Report . The relationship between biopharma R&D investment and expected returns: improving evidence to inform policy. Accessed May 6, 2024. https://itif.org/publications/2024/05/06/relationship-between-biopharma-rd-investment-expected-returns/

[3] Congressional Budget Office Letter to Chairman Arrington House Budget Committee . Re: Additional information about drug price negotiation and CBO's simulation model of drug development. Accessed December 21, 2023. https://www.cbo.gov/system/files/2023-12/59792-Letter.pdf

[4] Vital Transformation . IRA's impact on the US Biopharma Ecosystem, June 2023. Accessed September 30, 2025. https://vitaltransformation.com/2023/05/iras-impact-on-the-us-biopharma-ecosystem/.

[5] Patterson J, Motyka J, O'Brien JM. Unintended consequences of the Inflation Reduction Act: clinical development toward subsequent indications. Am J Manag Care. 2024;30(2):82–86. 10.37765/ajmc.2024.89495 38381543

[6] Charles River Associates Report . Implications of the Inflation Reduction Act for cancer medicine development. Accessed September, 2024. https://media.crai.com/wp-content/uploads/2024/09/25173656/Implications-of-the-Inflation-Reduction-Act-for-cancer-medicine-development-September2024.pdf

[7] Grabowski H, Long G. Post-approval indications and clinical trials for cardiovascular drugs: some implications of the US Inflation Reduction Act. J Med Econ. 2024;27(1):463–472. 10.1080/13696998.2024.2323903 38419523

[8] IQVIA Institute Report . Proliferation of innovation over time: frequency, timing and clinical value of expansions post-initial approval. Accessed February 18, 2025. https://www.iqvia.com/insights/the-iqvia-institute/reports-and-publications/reports/proliferation-of-innovation-over-time

[9] Centers for Disease Control and Prevention (CDC) . Chronic disease prevalence in the US: sociodemographic and geographic variations by zip code tabulation area, 2024. Accessed September 30, 2025. https://www.cdc.gov/pcd/issues/2024/23_0267.htm 10.5888/pcd21.230267 PMC10944638 38426538

